# Orbital Angular Momentum-based Space Division Multiplexing for High-capacity Underwater Optical Communications

**DOI:** 10.1038/srep33306

**Published:** 2016-09-12

**Authors:** Yongxiong Ren, Long Li, Zhe Wang, Seyedeh Mahsa Kamali, Ehsan Arbabi, Amir Arbabi, Zhe Zhao, Guodong Xie, Yinwen Cao, Nisar Ahmed, Yan Yan, Cong Liu, Asher J. Willner, Solyman Ashrafi, Moshe Tur, Andrei Faraon, Alan E. Willner

**Affiliations:** 1 Department of Electrical Engineering, University of Southern California, Los Angeles, CA 90089, USA; 2 T. J. Watson Laboratory of Applied Physics, California Institute of Technology, Pasadena, CA 91125, USA; 3 NxGen Partners, Dallas, TX75219, USA; 4 School of Electrical Engineering, Tel Aviv University, Ramat Aviv 69978, Israel

## Abstract

To increase system capacity of underwater optical communications, we employ the spatial domain to simultaneously transmit multiple orthogonal spatial beams, each carrying an independent data channel. In this paper, we show up to a 40-Gbit/s link by multiplexing and transmitting four green orbital angular momentum (OAM) beams through a single aperture. Moreover, we investigate the degrading effects of scattering/turbidity, water current, and thermal gradient-induced turbulence, and we find that thermal gradients cause the most distortions and turbidity causes the most loss. We show systems results using two different data generation techniques, one at 1064 nm for 10-Gbit/s/beam and one at 520 nm for 1-Gbit/s/beam; we use both techniques since present data-modulation technologies are faster for infrared (IR) than for green. For the 40-Gbit/s link, data is modulated in the IR, and OAM imprinting is performed in the green using a specially-designed metasurface phase mask. For the 4-Gbit/s link, a green laser diode is directly modulated. Finally, we show that inter-channel crosstalk induced by thermal gradients can be mitigated using multi-channel equalisation processing.

There is growing interest in high-capacity underwater wireless communications in order to support the significant increase in demand for data, such as from sensor networks, unmanned vehicles and submarines^1^,^2^,^3^,^4^,^5^. Traditionally, acoustic waves have been used for underwater communications, but this technique has quite limited bandwidth capacity^1^,^2^,^5^,^6^,^7^. Alternatively, optics in the low-attenuation blue-green region can enable higher-capacity underwater transmission links due to the much higher carrier-wave frequency^8^,^9^,^10^,^11^,^12^,^13^,^14^,^15^. In order to increase the capacity of underwater communications, a laudable goal would be to simultaneously transmit multiple independent data channels by using the spatial domain for multiplexing, i.e., space division multiplexing (SDM)^16^. If the beams are mutually orthogonal, the different beams can then be efficiently (de-)multiplexed, transmitted through a single transmitter/receiver aperture pair, and co-propagate with little inherent crosstalk.

An orthogonal spatial modal basis set that might enable underwater SDM is orbital angular momentum (OAM) modes^17^. A light beam with a helical wavefront carries an OAM value corresponding to 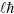 per photon, where 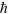 is the reduced Planck’s constant and 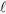 is an unbounded integer that represents the number of 2π phase changes in the azimuthal direction^17^,^18^. The phase front of an OAM beam twists along the propagation direction and results in a ring-shaped intensity profile with a central null^18^. Previous reports have explored the use of OAM multiplexing for high-capacity data transmission through the atmosphere using 1.55-μm light^19^,^20^,^21^,^22^,^23^. In general, free-space systems may need to deal with atmospheric turbulence, which can disrupt the beams’ phase fronts and cause intermodal crosstalk^24^,^25^,^26^,^27^.

Much has been uncovered in free-space OAM systems in the infrared (IR), yet little has been reported for underwater blue-green OAM communications. Indeed, the underwater environment presents several different challenges for a high-speed OAM link^28^,^29^. For example, the OAM beam itself and the data it carries can be significantly degraded due to various widely-varying effects, such as dynamic scattering/turbidity, water currents, and temperature gradients^12^,^13^,^14^,^15^,^30^,^31^,^32^,^33^,^34^. Although these issues are challenging for non-OAM, single beam underwater links, the problem may escalate for systems using phase front-sensitive OAM beams^25^,^33^,^34^.

Recent reports have shown a 4.8-Gbit/s underwater link using a conventional Gaussian beam by directly modulating a 1.2-GHz bandwidth 450-nm laser diode with orthogonal-frequency-division-multiplexing (OFDM) data^11^. Moreover, it has been shown recently that two multiplexed blue OAM beams each with a 1.5 Gbit/s data signal can propagate through 3-metre of water, which includes a scattering solution^28^. However, little has been reported on the performance degradation of OAM-multiplexed underwater data transmission due to other underwater effects (e.g., current and thermal gradients) as well as potential mitigation approaches to increase system performance.

In this paper, we explore OAM multiplexing for high-speed underwater communications, and we demonstrate up to a 40 Gbit/s link by transmitting four multiplexed green OAM beams through 1.2-metre of water^35^. Furthermore, we investigate the impact of various underwater conditions (e.g., scattering/turbidity, current, and thermal gradients) on beam quality and system performance, finding that thermal gradients can produce significant beam-quality degradation (e.g., modal distortion and beam wander). Importantly, we show systems results using four beams but with two different approaches for data modulation, one at 10-Gbit/s/beam in the infrared (IR) and one at 1-Gbit/s/beam in the green); we show both approaches since data modulation technologies are currently faster in the IR^5^,^36^. For the IR approach, we modulate a 1064-nm beam at 10-Gbit/s/beam and frequency double it into the green by using a periodically poled lithium niobate (PPLN) nonlinear crystal, and a specially designed integrated dielectric metasurface phase mask^37^ imprints the OAM on the beam; note that this 40-Gbit/s aggregate capacity is ~8 times higher than the previously reported result using a single conventional Gaussian beam^11^. For the green approach, we directly modulate the 532-nm laser diode and achieve a total capacity of 4 Gbit/s. Finally, in order to take advantage of the multiple beams traversing the same medium, we demonstrate that inter-channel crosstalk induced by thermal gradients can be mitigated using a multi-channel equalisation digital signal processing (DSP) algorithm at the receiver^38^.

## Results

**Figure 1:**
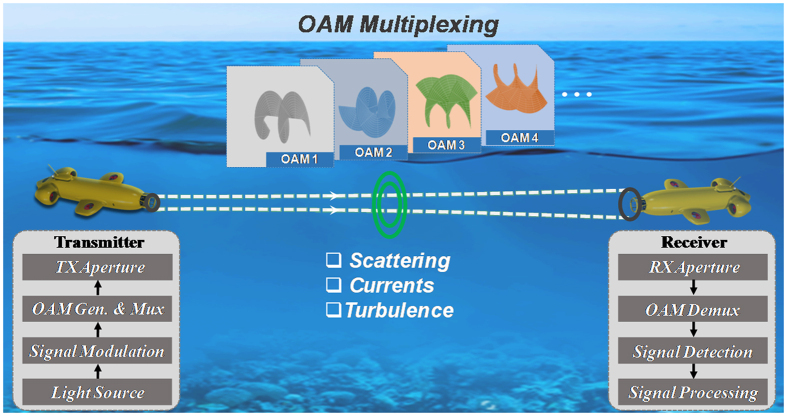
Prospective application scenario for a high-capacity underwater optical communications link with OAM-based space division multiplexing. Key modules including light source, signal modulation, OAM generation/multiplexing, OAM demultiplexing/detection and receiver signal processing are shown.

Figure 1 illustrates a prospective application scenario of using OAM multiplexing for high-speed underwater data transmission. We explore such a scenario under laboratory conditions to (a) help determine the challenges of OAM-based SDM underwater communications, (b) demonstrate ~8 times increase in underwater transmission capacity, and (c) show DSP-based mitigation of inter-channel crosstalk effects induced by thermal gradients.

### OAM beam propagation through various water conditions

We first investigate the influence of underwater propagation on green OAM beams. In general, a light beam propagating through water may suffer degradation from various effects, including scattering/turbidity, currents, and turbulence. We emulate these underwater conditions in a 1.2-metre-long rectangular tank (with 17 cm in width and 30 cm in height) filled with tap water. Specifically, underwater scattering/turbidity is produced with suspensions of Al(OH)_3_ and Mg(OH)_2_, which are obtained by adding a commercial antacid preparation (Maalox^®^)^8^,^12^,^14^. Circulation pumps pointing perpendicular to the propagation direction are evenly placed along the link path inside the water tank to produce a water current. Additionally, thermal gradient-induced water turbulence is created by introducing temperature inhomogeneity along the optical link, which is accomplished via mixing room temperature and heated water. The measured power loss induced by traversing the tank and the 1.2 metres of tap water is around 2.5 dB, which is mainly caused by the reflections at the tank’s glass interfaces.

**Figure 2:**
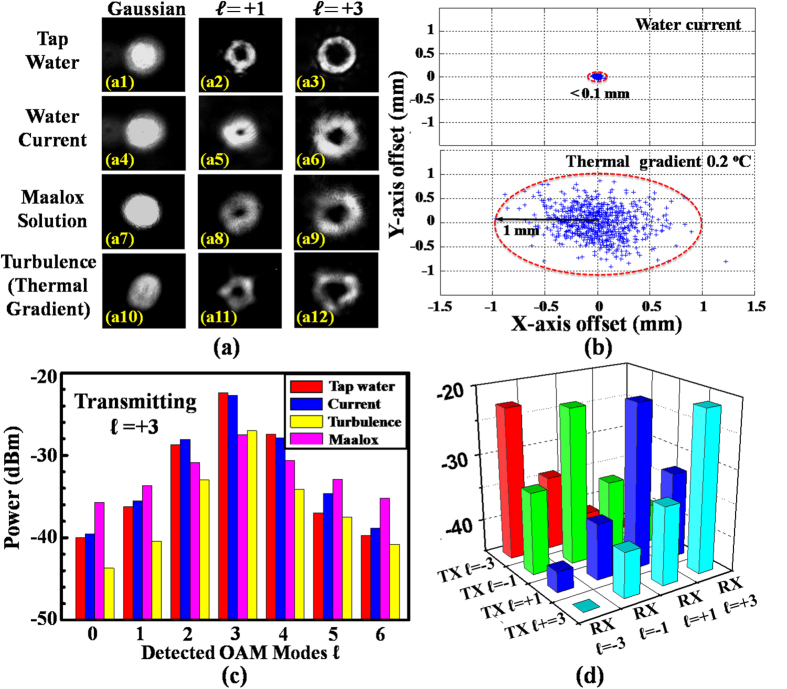
OAM beam propagation through various underwater channel conditions. (**a**) Intensity profiles of OAM beams under various channel conditions: (a1–a3) with only tap water, (a4–a6) with water current, (a7–a9) with the Maalox solution, and (a10–a12) with thermal gradient-induced turbulence. (**b**) Statistics for beam wander at the receiver with respect to the propagation axis due to water current and thermal gradient-induced turbulence. (**c**) OAM power spectrum when transmitting OAM channel 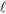 = +3 under various conditions. (**d**) Power transfer between all OAM channels under water current.

Figure 2(a) shows the intensity profiles of the individually transmitted and received Gaussian (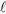 = 0) and OAM beams (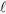 = +1 and +3) at 520 nm under various conditions: (i) only tap water (a1–a3), (ii) tap water with only current (a4–a6), (iii) tap water with only Maalox solution (a7–a9), and (iv) tap water with only thermal gradient (a10–a12). The OAM beam with either 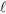 = +1 or +3 is generated by shining the Gaussian beam onto a spatial light modulator (SLM) loaded with a helical phase pattern of 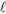 = +1 or 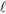 = +3. The water current is created using three circulation pumps, each with a flow rate of 26.8 litters per min. The Maalox solution added into the water tank containing 30 litters of water is 1.5-millilitre of 0.5% diluted Maalox, and the water after adding the Maalox is circulated by pumps for 1 minute to obtain a uniform scattering suspension (see Fig. S1(a,b) in Supplementary Section 1 for the case of a nonuniform suspension when there is no added water circulation). The room temperature and heated water that are mixed for turbulence emulation have a temperature difference of 0.2 °C; such an approach has been used previously to emulate thermal gradients in water^33^,^34^.

We observe that the ring-shaped intensity profiles of the OAM beams tend to be maintained after propagating through tap water, and are slightly distorted by water current, as shown in Fig. 2(a1–a6). When a 1.5-millilitre Maalox solution is added into still water, there is a small, time-varying change in the intensity profiles. This change might be a result of the natural dynamic diffusive movement of Al(OH)_3_/Mg(OH)_2_ particles in the water. However, when the particles become evenly distributed in the water after 1-minute circulation from one pump: (i) the distortions of the OAM intensity profiles tend to be small (Fig. 2(a7–a9)), and (ii) an additional power loss of 4.5 dB to the link is measured; note that the additional power loss is only 2.2 dB with a 0.5-millilitre of 0.5% diluted Maalox solution (see Supplementary Section 1). We expect a larger power loss for a higher concentration of scattering particles.

Figure 2(a10–a12) depict snapshots of intensity profiles under thermal gradient-induced turbulence, showing significant distortions in the beam profiles. We believe that this is mainly due to the higher-order wavefront aberrations that can result from the refractive index inhomogeneity induced by the water thermal gradient^33^,^34^. Moreover, the thermal gradient introduces a dynamic beam wander at the receiver, as depicted in Fig. 2(b). (obtained from 600 sequentially measured images). The maximal displacement of the received beams is estimated to be ~1 mm, which increases with a larger thermal gradient (as shown in Supplementary Fig. S1(c)). The beam wander (tip/tilt aberrations) combined with other higher-order wavefront aberrations (high-order Zernike polynomial terms) could cause the spreading of the transmitted OAM beam power into neighbouring modes, resulting in significant performance degradation (Supplementary Fig. S1(d)).

The beam wander plot due to water current is also provided in Fig. 2(b), showing a <0.1 mm beam displacement, which is much smaller than that with the thermal gradient. We believe this is because that water current introduces little effect on the homogeneity of water. Figure 2(c) shows the OAM power spectrum for beam 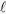 = +3 under the above underwater conditions. Due to the relatively small beam degradation caused by water current, the crosstalk value onto adjacent modes is less than 0.5 dB with only current. However, this value significantly increases to be >7 dB with thermal gradient-induced turbulence. Figure 2(d) presents the power transfer between OAM modes 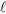 = ±1 and ±3 under water current. It is estimated that the total crosstalk for each mode is below −10.3 dB if all four beams are simultaneously transmitted.

The above measurements indicate that the thermal gradient-induced turbulence has a larger impact on beam quality than scattering or current, yet the Maalox-induced scattering if uniformly distributed may introduce significant link loss; additionally, non-uniform scattering may result in beam quality degradation.

### System performance measurements of four OAM multiplexed underwater links

In this section, we present the system performance measurements for simultaneously transmitting four OAM beams but using two different source data generation techniques. The first link transmits a 1-Gbit/s signal at 520 nm on each beam using the direct modulation of a laser diode, resulting in a capacity of 4 Gbit/s. For the second link, each beam carries a 10-Gbit/s signal generated using frequency doubling of a data signal at 1064 nm, achieving a significantly higher capacity of 40 Gbit/s.

#### 4-Gbit/s data link using directly modulated laser diodes

Two 1-Gbit/s on-off-keyed (OOK) signal beams at 520 nm are generated by directly modulating each of the two 520-nm green laser diodes. The two modulated green light beams are converted into two different OAM beams with 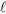 = +1 and +3 by adding different spiral phase patterns using SLMs. The generated OAM beams are coaxially combined and then split into two identical copies. Another two beams with opposite 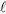 values of −1 and −3 can then be obtained by reflecting one of the copies three times. We note that this beam copy is relatively delayed with respect to the original one in free-space for data sequence decorrelation. Subsequently, the resulting four beams are spatially multiplexed and then propagated through the above-mentioned water conditions. At the receiver, each of the four OAM channels is sequentially demultiplexed using another SLM and detected using a high-sensitivity silicon avalanche photodiode (APD) with 1-GHz bandwidth. The detected signal is amplified, filtered and sent to a 1-Gbit/s receiver for bit-error rate (BER) measurements (see Supplementary Section 2 for implementation details).

**Figure 3:**
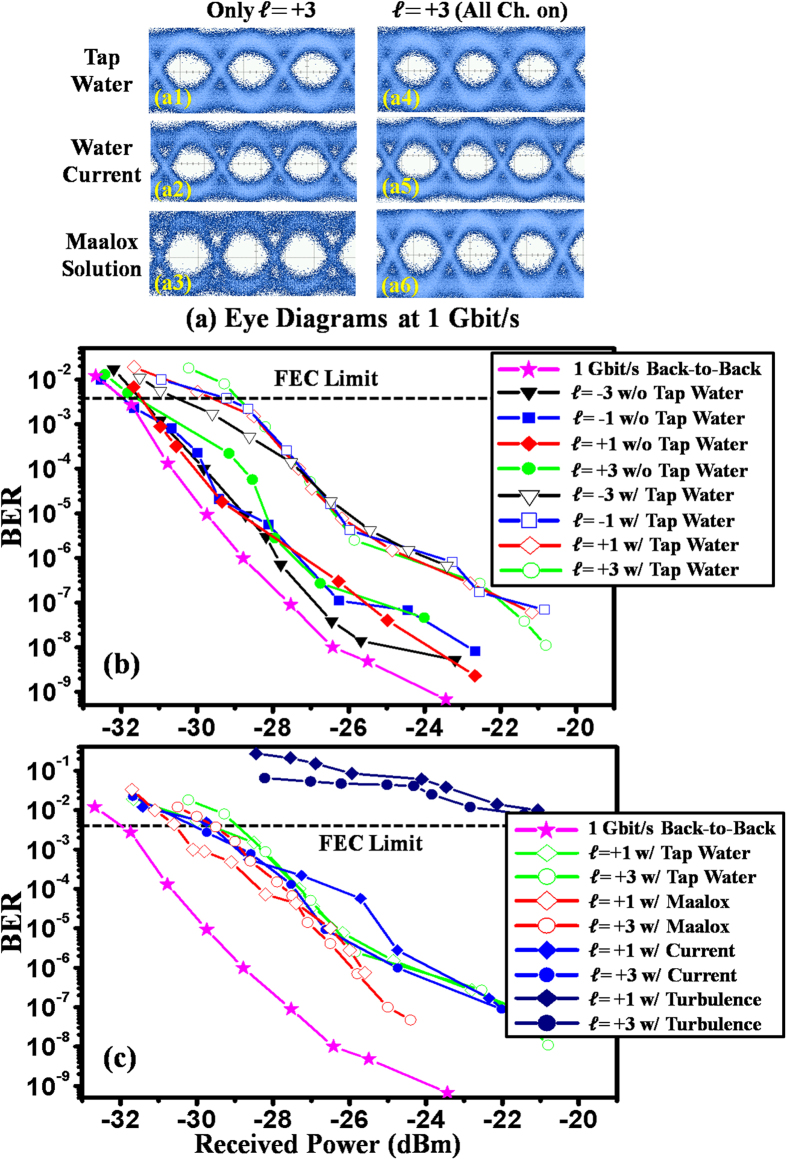
System performance measurements for the 4-Gbit/s underwater link using directly modulated laser diodes. (**a**) Eye diagrams for OAM channel 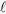 = +3, (**b**) BERs as a function of received power with and without tap water, and (**c**) BERs with Maalox-induced scattering, current and thermal gradient-induced turbulence.

Figure 3(a) depicts the eye diagrams of the 1-Gbit/s OOK signal for OAM channel 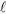 = +3 under various conditions when the other channels (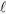 = −3, −1, and +1) are turned off or on. The inter-channel crosstalk effects can be clearly observed in Fig. 3(a4–a6). In the presence of a thermal gradient, the eye diagram of channel 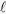 = +3 is time-varying due to fluctuations in the received power and crosstalk, and is not shown here. Figure 3(b) shows measured BERs as a function of received power for all four channels with and without water. The BER curve for the back-to-back (B2B) 1-Gbit/s signal is also provided as a benchmark. We observe that tap water introduces power penalties of less than 2.9 dB at the forward error correction (FEC) limit of 3.8 × 10^−3^ for all channels. Figure 3(c) presents BER curves for OAM channels 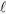 = +1 and +3 under various conditions. Power penalties are measured to be 2.2, 2.3, and 2.7 dB in the cases of tap water, Maalox-induced scattering and current, respectively. Due to the effects of thermal gradient-induced turbulence, the BERs are all above the FEC limit, exhibiting a severe error-floor phenomenon, and power penalties are above 12 dB for all channels.

#### 40-Gbit/s OAM link using PPLN-based frequency doubling

Due to water absorption, underwater optical communication links generally use blue-green light. However, data modulation technologies in this spectral region tend to have much lower bandwidths (e.g., around 1 GHz) than are available for IR light (e.g., beyond 10 GHz)^9^,^36^. An important goal would be the achievement of higher data rates for each underwater OAM channel. Therefore, modulating data in the IR region at a much higher speed and then wavelength converting it into the blue-green region for subsequent OAM generation and underwater transmission might enable significantly higher system capacities. Specifically, whereas we previously described data rates on each OAM beam of 1 Gbit/s, we show here the ability to transmit 10 Gbit/s on each beam using frequency doubling (see Supplementary Section 3 for implementation details).

A 10-Gbit/s OOK signal at 1064 nm is generated using a lithium niobate modulator and then amplified with a high power ytterbium-doped fibre amplifier (YDFA). The 1064-nm light after amplification is sent to a frequency-doubling module that consists of a PPLN crystal and a temperature stabilized crystal oven for frequency doubling. As a result, a 532-nm green light carrying a 10-Gbit/s data stream is generated, where its power depends upon both the oven temperature and the input pump power. The generated green light acts as a light source, being converted into OAM beams using specially-designed efficient dielectric metasurface phase masks^37^,^39^. Each phase mask has a diameter of 1.5 mm and is composed of a large number of square cross-section SiN_*x*_ nano-posts that locally modify the light’s phase with subwavelength spatial resolution. The nano-posts are 630-nm tall and are nested on a square lattice with the lattice constant of 348 nm. By changing the nano-post width in the range of 60 nm to 258 nm, the transmission phase of each point on the phase mask can be varied from 0 to 2π at 532 nm, based on which any arbitrary phase pattern can be designed. Phase masks of 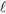 = ±1 and 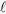 = ±3 each having a blazed grating ‘fork’ phase pattern (i.e., combination of the spiral phase structure of the desired OAM mode and a linear phase ramp^18^) are designed, fabricated, and characterized (see Supplementary Section 4). The fabricated phase masks are used to generate high-quality OAM beams with a 50% conversion efficiency (~3 dB).

Employing a setup similar to the one described in the previous section, the four OAM beams with 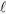 = ±1 and ±3 are spatially combined and propagate through the underwater channel. At the receiver, each of the four OAM data channels is sequentially demultiplexed using a metasurface phase mask with an inverse spiral phase pattern. The beam of the desired channel is spatially filtered after demultiplexing, detected using a high-bandwidth APD (3-dB cut-off frequency of 9 GHz) and sent to a 10-Gbit/s receiver for BER measurements.

**Figure 4:**
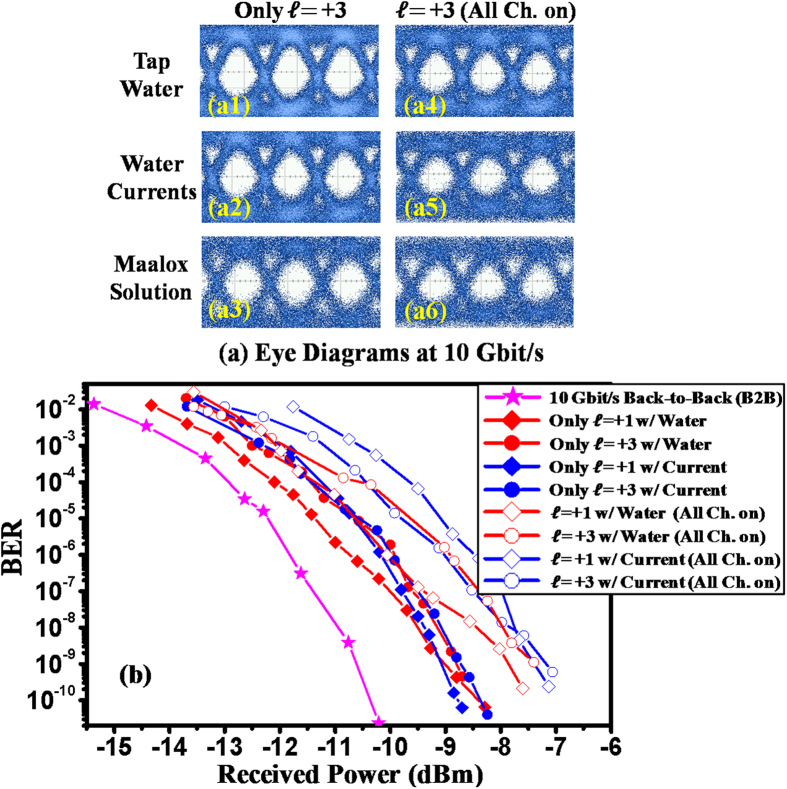
System performance measurements for the 40-Gbit/s underwater link using PPLN-based frequency doubling for signal generation. (**a**) Eye diagrams for OAM channel 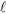 = +3 at a fixed transmitted power when other channels are turned off or on, and (**b**) BERs as a function of received power with tap water and current.

Figure 4(a) depicts the eye diagrams of the 10-Gbit/s OOK signal for OAM channel 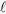 = +3 when the other channels are turned off and on. The total crosstalk from all the other channels are −11.2, −10.7, −11.0 dB for the cases of tap water, current, and Maalox scattering, respectively. Because of this, the quality of the eye diagrams degrades when other channels are turned on. Figure 4(b) shows measured BER curves for OAM channels 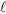 = +1 and +3 in the cases of tap water and current with and without crosstalk from the other channels. The B2B BER curve of the 10-Gbit/s signal is also provided. The power penalties are observed to be less than 2.2 dB for all cases when all channels are on.

### Mitigation of thermal gradient-induced crosstalk using multi-channel equalisation

Previous sections found various OAM beam degradations and consequent data-channel crosstalk based on underwater effects. In this section, we address the data degradation problem and show the mitigation of inter-channel crosstalk due to thermal gradient-induced turbulence. We employ a constant modulus algorithm (CMA)-based multi-channel equalisation in the receiver DSP to reduce channel crosstalk effects and thus recover the transmitted data streams^40^,^41^,^42^. This approach has been previously employed in few-mode and multi-mode fibre-based mode division multiplexed systems to mitigate the mode coupling effects among multiple spatial modes^43^,^44^. In general, it is required that all the transmitted channels are simultaneously detected to enable multi-channel equalisation processing. Due to receiver hardware limitations, we only show crosstalk mitigation between two OAM channels.

With a similar system approach, two OAM beams with 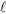 = +1 and +3 are generated using metasurface phase masks, spatially combined using a beam splitter and transmitted through water with a thermal gradient of 0.2 °C. Each OAM beam carries a 10-Gbit/s OOK signal generated by doubling the frequency of a modulated 1064-nm signal using a PPLN nonlinear crystal. After demultiplexing and detection, the two OAM channels are simultaneously received, converted into Gaussian-like beams and detected by two 9-GHz bandwidth APDs. The two signals are then amplified, sampled by a real-time scope and recorded for offline DSP. A 2 × 2 CMA equalisation algorithm is implemented in the DSP to recover two OAM data channels with 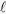 = +1 and 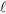 = +3. For a 2 × 2 CMA equalisation, the equaliser includes four adaptive finite-impulse-response (FIR) filters each with a tap number of 11, the coefficients of which can be adaptively updated until convergence based on the CMA algorithm (see Supplementary Section 5). The obtained FIR filter coefficients are used to equalise the crosstalk between the two OAM channels.

**Figure 5:**
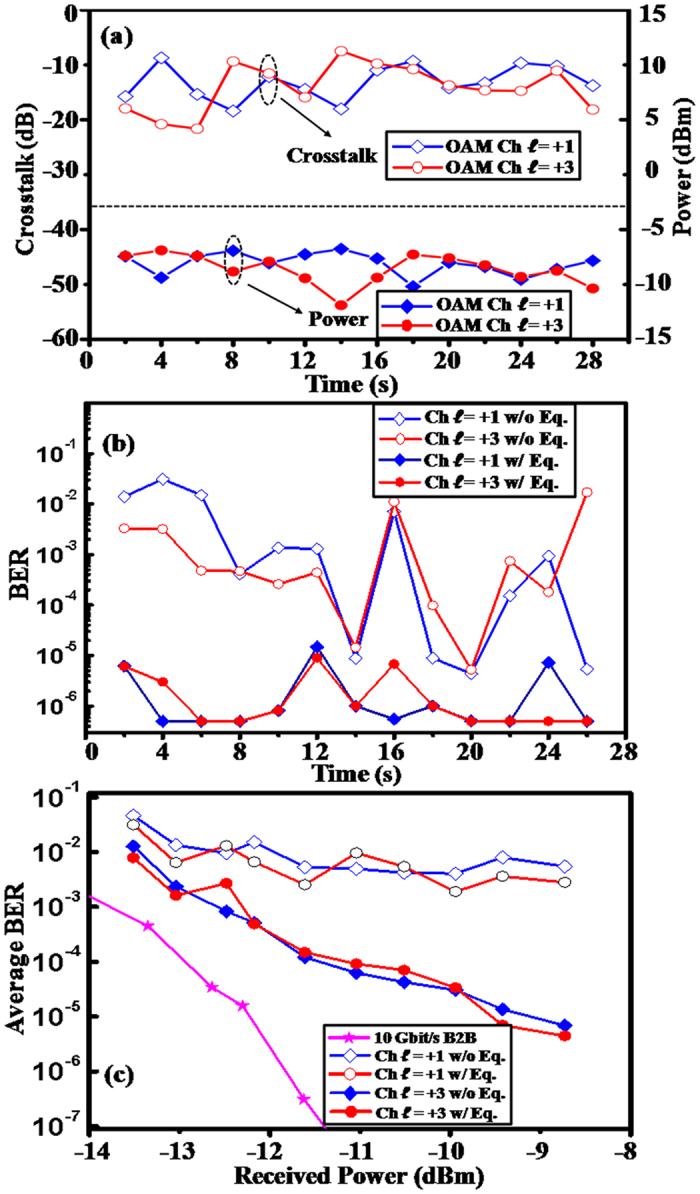
Mitigation of thermal gradient-induced crosstalk using CMA-based multi-channel equalisation. (**a**) Received power and channel crosstalk of OAM 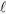 = +1 and 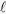 = +3 over 28 seconds and (**b**) instantaneous BER of OAM 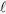 = +1 and 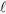 = +3 over 28 seconds with and without CMA equalisation under thermal gradient-induced turbulence when both channels are transmitted. (**c**) Measured BER curves of OAM channel 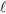 = +1 and 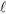 = +3 with and without CMA equalisation. Ch.: channel. Eq.: equalization.

Figure 5(a) depicts the received power and crosstalk of OAM channels 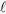 = +1 and +3 measured every 2 seconds under the effects of thermal gradient-induced turbulence. The received power and crosstalk fluctuate by up to 4.5 and 12.5 dB, respectively. The corresponding BERs for the two OAM channels during the same time period are shown in Fig. 5(b). Without CMA equalisation, the measured BERs fluctuate significantly between 1.7 × 10^−2^ and 7.4 × 10^−6^, and dramatically decrease, reaching below the FEC limit of 3.8 × 10^−3^ after 2 × 2 CMA equalisation. We note that only a length of 2,000,000 symbols is recorded for each data sequence due to the limited memory of the real-time scope, and therefore the minimum BERs that can be measured are around 5 × 10^−7^. To further illustrate the improvement, Fig. 5(c) shows the measured BERs averaged over 1 minute as a function of received power for channels 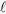 = +1 and +3. Due to inter-channel crosstalk, the measured BER curves without 2 × 2 equalization also have BER error floors. The power penalties at the FEC limit, compared to the B2B case, are below 2.0 dB for the two channels after equalisation.

## Discussion

The experiments described in this paper explore the potential of using OAM-based SDM to increase the transmission capacity of underwater optical communications, and several issues lend themselves to further exploration.

In general, the use of OAM multiplexing may require a more precise alignment between the transmitter and receiver compared to a single-channel underwater optical link. This is due to the fact that orthogonality among OAM channels relies on a common optical axis, and any misalignment may result in inter-channel crosstalk^45^. Given the beam wander that is introduced by thermal gradients, the above problem is exacerbated and may require an accurate pointing and tracking system.

Additionally, given that small thermal gradients can produce system degradation, we assume that this problem could become more severe for longer links for which different types of water may exist. Moreover, this problem may depend on the transmission direction, such that a vertical link may experience a different thermal gradient than a horizontal link. Furthermore, whereas we used a channel equalisation algorithm to help mitigate the thermal gradient-induced crosstalk, it might be necessary under harsher and wider-ranging underwater conditions to explore the use of multiple mitigation techniques^46^,^47^,^48^. We emphasise that other effects, such as spatial dispersion and object obstructions, are not considered in this paper but might cause beam spreading and link outage^49^,^50^.

Finally, we investigated the effects of underwater propagation on OAM-multiplexed data transmission and the mitigation of inter-channel crosstalk over a short link of metre-length scale. However, we believe our results could potentially be expanded to longer distances and scaled to a larger number of OAM channels through both careful system design^45^ and the use of multiple mitigation approaches for channel degradation effects. The potential transmission distance of an OAM multiplexed underwater link is limited by various factors, including thermal gradients, transmitter/receiver aperture size, and attenuation caused by water absorption; such attenuation depends critically on water type, e.g., coastal water, and oceanic water^3^,^12^. If we assume that the OAM beams are fully collected by the receiver aperture and the thermal gradients are mostly compensated by signal processing, we believe it could be possible to achieve a link distance over 50 metres given a link power loss of ~32 dB for a water type with an attenuation coefficient of 0.15 m^−1^. OAM multiplexing is in principle compatible with advanced modulation formats (e.g., quadrature-amplitude-modulation and OFDM) and wavelength division multiplexing. Particularly, beams with the same wavelength in the blue-green region can be reused by applying different OAM values to each of the many beams. When coupling the advances with implementing those techniques, we envision that the underwater transmission capacity of 40 Gbit/s achieved in this paper could be extended by an order-of-magnitude.

## Methods Summary

### Generation and detection of data-carrying green OAM beams

Two different data-modulation approaches are employed to generate high-speed green light signals:

#### 1-Gbit/s signal generation at green using internal modulation

By directly modulating the driving current of a 520 nm laser diode, a 1-Gbit/s signal at 520 nm is produced. Due to the bandwidth limitation of the internal modulation of the laser diode, the maximal data rate of the green beam is 1 Gbit/s. The generated signal is then launched onto a programmable SLM with a specific helical phase pattern to create an OAM beam with either 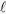 = +1 or +3. Multiple generated OAM beams are then multiplexed using a beam splitter-based combiner and the resulting beams propagate through the underwater channel. The received signal after demultiplexing is detected using a high sensitivity Si APD with a 3-dB bandwidth of 1-GHz.

#### 10-Gbit/s signal generation at green using PPLN-based frequency doubling

Generally, the modulation bandwidth of both internal and external modulations for green light is limited to GHz^4^,^7^. To overcome this, the frequency doubling of a data-carrying 1064-nm signal is thus used to produce a high-speed green light signal. Specifically, we perform high-speed data modulation using a 1064-nm lithium niobate modulator and use a PPLN-based frequency-doubling module to convert the carrier wavelength from 1064 nm to 532 nm. Consequently, a green light signal at 532 nm is generated, which is then split into multiple copies and converted into OAM beams using transmissive metasurface phase masks. At the receiver, a Si APD with a 3-dB bandwidth of 9-GHz but a lower sensitivity than the detector used for the 520-nm signal detection is employed for signal detection.

### Crosstalk mitigation using multi-channel equalisation

The multiplexed OAM beams may be distorted due to underwater propagation, causing the power spreading of each transmitted OAM mode onto neighbouring modes. Consequently, each OAM channel experiences interferences from the other channels, resulting in a non-diagonal channel matrix. Theoretically, to recover the data streams, the received signals of all OAM channels could then be multiplied with the inverse channel matrix. In our experiment, we use a 2 × 2 CMA adaptive channel equalisation in the receiver to reduce the effects of interferences and recover the two data channels. In general, the dimension of the equalisation processing is determined by the total number of OAM channels. The CMA-based equalisation utilises an FIR filter-based linear equaliser for each channel. The FIR-CMA equaliser contains four FIR filters, the coefficients of which can be adaptively updated until convergence based on the CMA. The obtained FIR filter coefficients are used to equalise the crosstalk between two OAM channels.

## Additional Information

**How to cite this article**: Ren, Y. *et al*. Orbital Angular Momentum-based Space Division Multiplexing for High-capacity Underwater Optical Communications. *Sci. Rep*. **6**, 33306; doi: 10.1038/srep33306 (2016).

## Supplementary Material

Supplementary Information
