# Supplementary material for: Orbital Angular Momentum-based Space Division Multiplexing for High-capacity Underwater Optical Communications
Source: Sci Rep. 2016 Sep 12;6:33306. doi: 10.1038/srep33306 (PMC5018855; doi:10.1038/srep33306)
Supplement: Supplementary Information [file srep33306-s1.pdf]

Orbital Angular Momentum-based Spatial Division Multiplexing for High-capacity Underwater Optical Communications

Yongxiong Ren[1]*, Long Li[1]*, Zhe Wang[1], Seyedeh Mahsa Kamali[2], Ehsan Arbabi[2], Amir Arbabi[2], Zhe Zhao[1], Guodong Xie[1], Yinwen Cao[1], Nisar Ahmed[1], Yan Yan[1], Cong Liu[1], Asher J. Willner[1], Solyman Ashrafi[3], Moshe Tur[4], Andrei Faraon[2], and Alan E. Willner[1]

[1]Department of Electrical Engineering, University of Southern California, Los Angeles, CA 90089, USA.

[2]T. J. Watson Laboratory of Applied Physics, California Institute of Technology, Pasadena, CA 91125, USA

[3]NxGen Partners, Dallas, TX75219, USA

[4]School of Electrical Engineering, Tel Aviv University, Ramat Aviv 69978, Israel.

*These authors contributed equally to this work.

1. OAM beams underwater propagation: Measurement results under different scattering levels and thermal gradients

We show below measurements of OAM beams propagation under a different scattering level and thermal gradient. Supplementary Figs. S1(a1–a2) show BER fluctuations over 36 seconds at a fixed transmitted power of -26 dBm when different amounts of 0.5% diluted Maalox solution are added into the water. These measurements are performed every 2 seconds and are repeated 18 times before the Maalox particles are evenly distributed in the water (before circulation over 1 minute). We see that the case of 0.5-millilitre of Maalox solution has a larger BER fluctuation range due to a more uneven suspension of Maalox particles along the link path. After uniform

scattering suspension is obtained, the 0.5- and 1.5-millilitre Maalox solutions introduce power losses of 2.2 dB and 4.5 dB to the link, respectively. Fig. S1(c) presents the statistics of beam displacement with respect to the propagation axis after propagation through thermal gradient-induced turbulence, in which the room temperature water and the heated water have a temperature difference of 0.3^{o}C. We see that the received beams' maximal displacement is estimated to be 1.4 mm, which is larger than that shown in Fig. 2(b) of the main manuscript. The captured intensity profiles of OAM beam ℓ=+3 under 3 different turbulence realisations are also shown to illustrate the time-varying distortions caused by dynamic turbulence. Fig. S1(d) shows the BERs and received power for the OAM ℓ =+3 channel under various turbulence realisations. A wider range of power fluctuation is observed than that shown in Fig. 5(a) of the main manuscript.

2. Implementation details of the 4 Gbit/s OAM multiplexed underwater optical link using directly modulated green laser diodes

In this section, we describe the experimental implementation of the 4 Gbit/s four OAM multiplexed underwater link, in which laser diodes at 520 nm are directly modulated and act as light signal sources. Supplementary Fig. S2 presents the link schematic. Two 1-Gbit/s OOK signal beams at 520 nm are generated by directly modulating each of the two 520-nm green laser diodes with a binary sequence. The two modulated green light beams are launched onto two liquid crystal-based spatial light modulators (SLMs) to create two different OAM beams with ℓ=+1 and +3. The SLM (Santec Inc.) has a pixel resolution of 1440×1050 and an operating wavelength range of 500-1650nm. These two OAM beams ℓ=+1 and +3 are coaxially combined and then split into two identical copies, one of which is reflected three times using mirrors arranged to introduce a ~50 ns delay for data sequence decorrelation between the two copies.

Another two OAM beams with opposite ℓ values of-1 and -3 are obtained due to the odd number of reflections, and these new beams are then combined with the original OAM beams ℓ = +1 and +3 by a beam splitter. The resulting four multiplexed OAM beams propagate through the underwater channel.

(a) Multiplexed OAM beams at the transmitter

dBm	ℓ= -3	ℓ= -1	ℓ= +1	ℓ= +3
**ℓ= -3**	-23.72	-34.12	-40.82	-44.53
**ℓ= -1**	-35.08	-21.42	-36.52	-39.46
**ℓ= +1**	-40.60	-32.89	-21.78	-33.06
**ℓ= +3**	-40.13	-38.43	-32.58	-20.91

(b) After propagating through tap water

dBm	ℓ= -3	ℓ= -1	ℓ= +1	ℓ= +3
**ℓ= -3**	-23.39	-34.01	-39.90	-42.87
**ℓ= -1**	-36.80	-21.89	-32.45	-39.23
**ℓ= +1**	-39.20	-34.12	-22.67	-34.50
**ℓ= +3**	-42.80	-39.26	-34.89	-22.35

Table 1. The power transfer between the four multiplexed OAM channels (a) at the transmitter and (b) after propagating through tap water.

We characterize the power leakage and crosstalk between all OAM channels. The power leakage is measured in the following way: We first transmit a 520-nm signal over OAM channel ℓ=-3 while all the other channels (OAM beams ℓ=-1, +1, and +3 are off. Then we record the power leaked into other OAM modes (OAM channels ℓ =±1 and ±3). The above measurements are repeated for all transmitted OAM channels until a full 4×4 power transfer matrix is obtained. Table 1 shows the power transfer matrices between all the four channels at the transmitter and after propagating the tap water channel. The crosstalk of a specific channel can be calculated from the 4×4 power transfer matrix by adding the received power from all other channels divided

by the received power of this channel. We see that the crosstalk values for all four multiplexed channels are below -10 dB in both cases, and the degradation introduced by tap water is negligible.

Three different underwater channel conditions are emulated in a 1.2-metre-long rectangular tank filled with tap water, as described in the main manuscript. The water current and scattering are created by distributed circulation pumps and the Maalox solution, respectively. The thermal gradient-induced turbulence is produced by mixing the room temperature and heated water. After propagating through the various water conditions, the four multiplexed OAM channels are sequentially demultiplexed and detected at the receiver. To recover the data channel carried by OAM beam $+\ell$, the SLM used for demultiplexing is loaded with a inverse spiral phase pattern of -ℓ. As a result, only OAM beam with $+\ell$ is converted into a Gaussian-like beam ($\ell = 0$) and all the other OAM beams maintain ring-shaped profiles, which can be efficiently filtered out by a spatial filter (simply a pin hole). The Gaussian-like beam is subsequently focused onto a high-sensitivity silicon avalanche photodiode detector (APD) with a 3-dB bandwidth of 1 GHz. The APD has a spectral responsivity of ~15 A/W at 520 nm and a low noise equivalent power (NEP) of 0.4 $pW/\sqrt{HZ}$. The signal after detection is amplified, filtered and sent to a 1-Gbit/s receiver for bit-error rate (BER) measurements.

3. Implementation details of the 40 Gbit/s OAM multiplexed underwater optical link using PPLN-based frequency doubling

We present below the implementation details of the 40 Gbit/s four OAM multiplexed underwater link, in which each 532-nm green OAM beam carries a 10 Gbit/s OOK signal generated from PPLN-based frequency doubling. The experimental setup is depicted in Supplementary Fig. S3.

A 1064-nm single-mode laser with a linewidth of less than 100 MHz is sent to a Lithium Niobate modulator to produce a 10 Gbit/s on-off keying (OOK) signal. The transmitted RF signal is a pseudorandom binary sequence of a length 2^{15}-1. The 10 Gbit/s OOK signal at 1064 nm is amplified by a ytterbium-doped fibre amplifier (YDFA) and fed into a collimator to convert the single-mode fibre output to a collimated Gaussian beam with a diameter of 2.6 mm. This Gaussian beam is then focused into the centre of a periodically poled lithium niobate (PPLN) crystal for frequency doubling. A half-wave plate is inserted after the collimator to align the polarisation of incident light with the polarisation orientation of the PPLN crystal to maximise the conversion efficiency. The PPLN crystal is z-cut to 20 mm × 1 mm × 1 mm in dimension. Both its input and output facets are dual-coated with reflectivity of <1% at 532 nm and 1064 nm to reduce the Fresnel reflection loss. The crystal oven together with a temperature controller offers a stability of ±0.01^{o}C. The power of the green beam generated from frequency doubling depends upon both the temperature and the input pump power. For a 34 dBm 1064-nm input beam, the power of the output green beam at 532 nm is around 22 dBm with the oven temperature being 79.5 ^{o}C. The beam after the PPLN crystal is collimated and passes through a dichroic mirror to reflect the unconverted 1064-nm beam. A bandpass filter with a centre wavelength of 532 nm is followed to further separate the green light from the remaining 1064-nm beam. As a result, a 532-nm green beam carrying a 10 Gbit/s OOK signal is produced. We note that this frequency-doubling process is transparent to intensity-based modulation format.

The resulting signal beam at 532 nm is split into two copies, which then pass through two transmissive metasurface phase masks to generate two OAM beams with ℓ=+1 and +3, respectively. The metasurface phase masks have high efficiency (power loss of ~3dB at 532 nm) and the generated OAM modes are of high quality (see Supplementary Note 4 for more details

about the design). These two OAM beams are spatially combined using a beam splitter. Using a similar approach as in the previous section, another two OAM beams with opposite ℓ values (ℓ = -1 and -3) can be obtained, which are then multiplexed with the original OAM beams ℓ = +1 and +3. The beam sizes for OAM beams ℓ = -3, -1, +1 and +3 are 2.1, 1.5, 1.4 and 2.0 mm, respectively. The resulting four multiplexed OAM beams (ℓ = ±1 and ±3) are sent through a 1.2-metre underwater channel. The power transfer matrices between the four channels at the transmitter and after propagating the tap water channel are shown in Table. 2. We see that the crosstalk values for all four channels are below -11 dB in both cases.

(a) Multiplexed OAM beams at the transmitter

dBm	ℓ= -3	ℓ= -1	ℓ= +1	ℓ= +3
**ℓ= -3**	-17.3	-29.8	-31.2	-39.6
**ℓ= -1**	-31.5	-17.4	-28.3	-38.74
**ℓ= +1**	-38.7	-30.9	-17.5	-28.3
**ℓ= +3**	-17.7	-32.3	-29.2	-17.7

(b) After propagating through tap water

dBm	ℓ= -3	ℓ= -1	ℓ= +1	ℓ= +3
**ℓ= -3**	-19.99	-31.8	-33.65	-40.18
**ℓ= -1**	-32.07	-20.1	-31.66	-37.42
**ℓ= +1**	-35.49	-30.1	-19.89	-33.12
**ℓ= +3**	-40.18	-35.1	-30.6	-19.83

Table 2. The power transfer between the four multiplexed OAM channels (a) at the transmitter and (b) after propagating through the tap water channel.

After propagation through water, a phase mask with an inverse phase pattern of the desired OAM channel will be used to convert the chosen OAM beam into a Gaussian like beam. The other beams maintain their ring-shaped profiles and helical phases after passing through the phase mask. The Gaussian like beam has a bright high intensity at the beam centre and is thus separable

from the other beams through spatial filtering (simply a pin hole). This beam is then focused onto a 9-GHz-bandwidth silicon APD, which has a spectral responsivity of ~0.2 $\mathrm{A/W}$ at 532 nm and a NEP of <45 $\mathrm{pW}/\sqrt{\mathrm{HZ}}$. The signal after the APD is amplified, filtered and sent to a 10 Gbit/s OOK receiver for BER measurements. Given that four OAM beams each bearing a 10 Gbit/s data stream are transmitted, a total capacity of 40 Gbit/s is achieved.

Supplementary Figs. S4(a1–a4) show the captured intensity profiles of the generated OAM beams of ℓ = ±1 and ℓ = ±3 at the transmitter. Figures. S4(b1–b2) presents the measured interferograms for OAM beams ℓ = +1 and +3, in which the state number of the two OAM beams can be deduced from the number of rotating arms. Each interferogram can be obtained interfering an OAM beam (either ℓ = +1 or +3) with an expanded Gaussian beam. Figs. S4(c1–c4) show the measured intensity profiles (c1-c2) and interferograms (c3-c4) of the received OAM beams ℓ = +1, and ℓ = +3 after propagating through tap water. Figs. S4(d1–d4) depict the intensity profiles of demultiplexed beams at the receiver when only the OAM channel ℓ = +3 is transmitted. We see that only when the phase mask is of inverse spiral phase of ℓ = -3, can the OAM beam with ℓ = +3 be converted into a Gaussian like beam with a high intensity at the beam centre.

4. High-efficiency dielectric metasurface OAM generator for visible wavelengths

In this section, we describe the design, fabrication and characterization of dielectric metasurface phase masks for OAM generation. Dielectric metasurface phase masks made of square cross-section SiN_x nano-posts on fused silica are designed, fabricated and characterised for the generation and detection of OAM beams at 532 nm. Polarisation-insensitive phase masks are composed of 630-nm-tall SiN_x nano-posts on a square lattice with the lattice constant of 348 nm.

By changing the nano-posts width in the range of 60 nm to 258 nm, the transmission phase can be changed from 0 to 2π while maintaining high transmissivity at the wavelength of 532 nm. Therefore, any arbitrary phase profile can be designed using this metasurface platform. Here, a blazed grating 'fork' phase pattern, which is a combination of the helical phase structure of the desired OAM mode and a small linear phase ramp (~2 degrees), is designed. Such a combination pattern can help separate the generated OAM beam from the residual unmodulated Gaussian beam, providing high-quality OAM generation and detection. Supplementary Fig. S5(a) shows a schematic illustration of the blazed grating 'fork' phase mask generating the OAM mode with ℓ=+3. The metasurfaces are fabricated by depositing a 630-nm thick layer of SiN_x on a fused silica substrate. The pattern is defined and transferred to the SiN_x layer using e-beam lithography followed by the lift-off process and dry etching. Two different 1.5-mm diameter metasurface phase masks with ℓ=+1 and ℓ=+3 are designed and fabricated. Figs. S5(b1–b2) show the scanning electron micrographs of the fabricated phase masks of ℓ=+1 and ℓ=+3, respectively. To quantify the quality of OAM beam generation, we measure the OAM power spectrum of generated OAM beams ℓ = +1 and ℓ = +3, as shown in Fig. S5(c). We see that the majority of the power resides in the desired modes and the power leakage onto the adjacent modes is -13 dB less than the desired modes.

5. Implementations details of the CMA-based multi-channel equalisation algorithm

In general, for an underwater optical link using M multiplexed OAM beams, a multiple-input multiple-output (MIMO) channel processing with a dimension of $M \times M$ would be needed to reduce the inter-channel crosstalk caused by underwater propagation (mainly thermal gradient-induced turbulence in the experiments). Mathematically speaking, the received signal vector for all M OAM channels $\boldsymbol{y} = (y_1, y_2, \dots, y_M)^{\mathrm{T}}$ can be expressed as

$$\boldsymbol{y} = \boldsymbol{Hx} + \boldsymbol{N} \quad (1)$$

where y_i $(i = 1,2, \ldots M)$ is the received signal of OAM channel i and $\boldsymbol{x} = (x_{1,} \, x_{2,} \, \ldots, x_M)^T$ with x_i being the transmitted signal for OAM channel i. $\boldsymbol{H}$ is the channel matrix, which can be written as

$$\boldsymbol{H} = \begin{bmatrix} h_{1,1} & h_{1,2} & \cdots & h_{1,M} \\ h_{2,1} & h_{2,2} & \cdots & h_{2,\mathrm{M}} \\ \vdots & \vdots & \ddots & \vdots \\ h_{M,1} & h_{M,2} & \cdots & h_{M,M} \end{bmatrix}_{M \times M}, \quad (2)$$

where $h_{i,j}$ depicts the transfer function between OAM channel i to OAM channel j. $\boldsymbol{N} = (n1, \; n2, \; \ldots, nM \;)T$ and ni is the noise for *i*-th OAM channel. $\boldsymbol{H}$ is determined by the power loss of each OAM channel and crosstalk between all M OAM channels, which are directly related to the underwater channel conditions and system design (e.g. aperture sizes and link distance). To recover the OAM data streams, the received signals of all OAM channels could then be multiplied with the inverse of channel matrix $\boldsymbol{H}$ theoretically.

A variety of implementation approaches for MIMO processing based on $\boldsymbol{H}$ have been proposed, including joint maximum likelihood sequence estimation of the data symbols in different streams, minimum mean-square error detection combined with serial interference cancellation, zero forcing detection, and pilot-aided channel equalisation. For the experiment using two multiplexed OAM modes, a 2×2 CMA-based equalisation algorithm is implemented to equalise crosstalk between the two OAM channels, thus allowing data recovery. After demultiplexing, each of the two received OAM channels is converted into a Gaussian-like beam and detected by a 1-GHz bandwidth APD. The two signals are amplified, sampled by a real-time scope and recorded for offline DSP. Given that we use the OOK modulation format for the signal

generation, which does not have a constant modulus in the signal constellation, the DC components of the two received channels are subtracted to apply the CMA algorithm. The CMA-based equalization algorithm utilises a linear equaliser for each channel. For a 2×2 equalisation, the equaliser includes 4 adaptive finite-impulse-response (FIR) filters, each with a tap number of K. Specifically, the output of the equaliser corresponding to each channel can be expressed as:

$$y_j = \sum_{i=1}^{2} \boldsymbol{w_{ij}} * \boldsymbol{x_i}, j = 1, 2 \tag{3}$$

where $\boldsymbol{w_{ij}}$ (i =1,2) is the coefficient vector of the FIR filter with a vector length of K (tap number). $\boldsymbol{w_{ij}} * \boldsymbol{x_i}$ represents the inner product operation between two vectors and y_j is the output of the FIR filter. All the FIR coefficients are initialised as zero with only the centre weight being 1 and then updated until the coefficients converge based on CMA:

$$\boldsymbol{w_{ij}}(k+1) = \boldsymbol{w_{ij}}(k) + u \cdot e_i \cdot y_i \cdot \boldsymbol{x_i}^* \tag{4}$$

where u is the step size, $e_i = P_{ref} - |y_i|^2$ is the error signal of the adaptive estimation and P_{ref} is the normalised reference power. The main idea of CMA-based MIMO equalisation is to update filter weights such that each channel output can have a clear amplitude. The tap number K in each FIR filter is set to be 11, which is sufficient to cover the differential time delays among each data sequence and mitigate temporal ISI effects. The obtained FIR filter coefficients are used to equalise the crosstalk among two OAM channels of Equation (3). After equalisation, the bit-error rates (BERs) are evaluated for both channels.

Supplementary Figures

Supplementary Figure S1

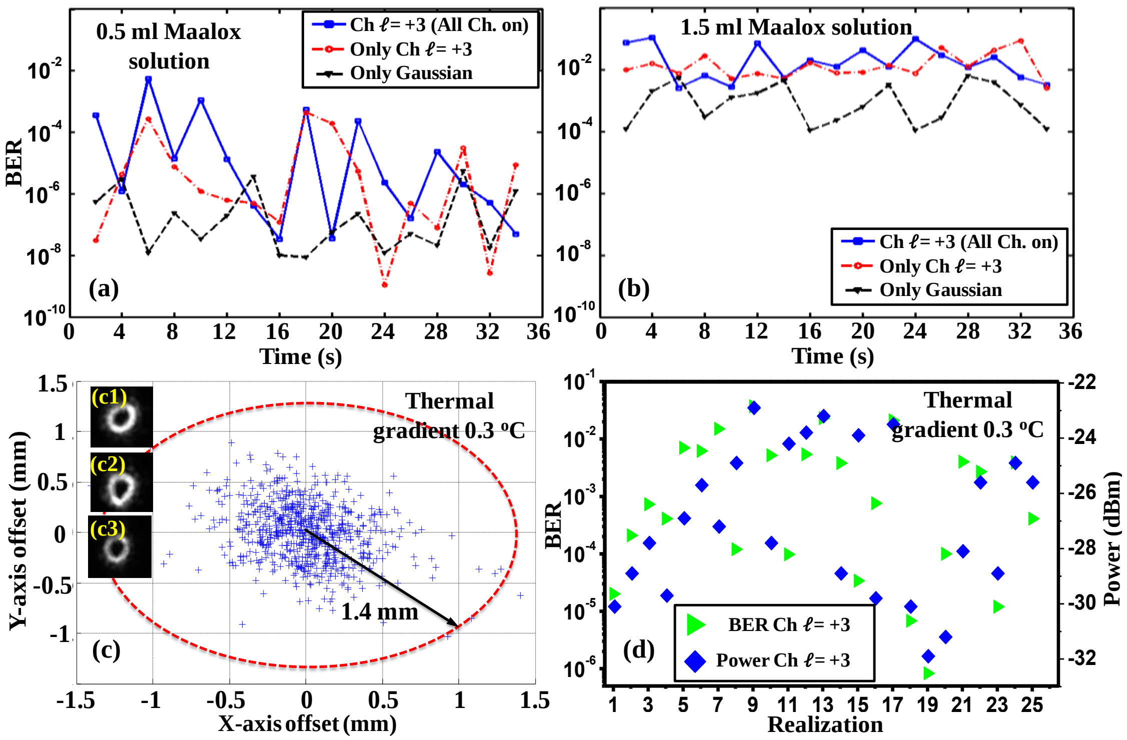

Supplementary Figure S1 | Measured signal fluctuations and beam wander at the receiver under different scattering conditions and thermal gradients. Instantaneous BERs of channel ℓ = +3 over 36 seconds at a fixed transmitted signal power of -26 dBm when (a) 0.5-millilitre and (b) 1.5-millilitre of Maalox solution are added into tap water (before 1-minute circulation). (c) Statistics for the displacement of the received beams with respect to the propagation axis due to thermal gradient-induced turbulence. (d) BERs and received power for the OAM ℓ =+3 channel under various realisations of thermal gradient-induced turbulence.

Supplementary Figure S2

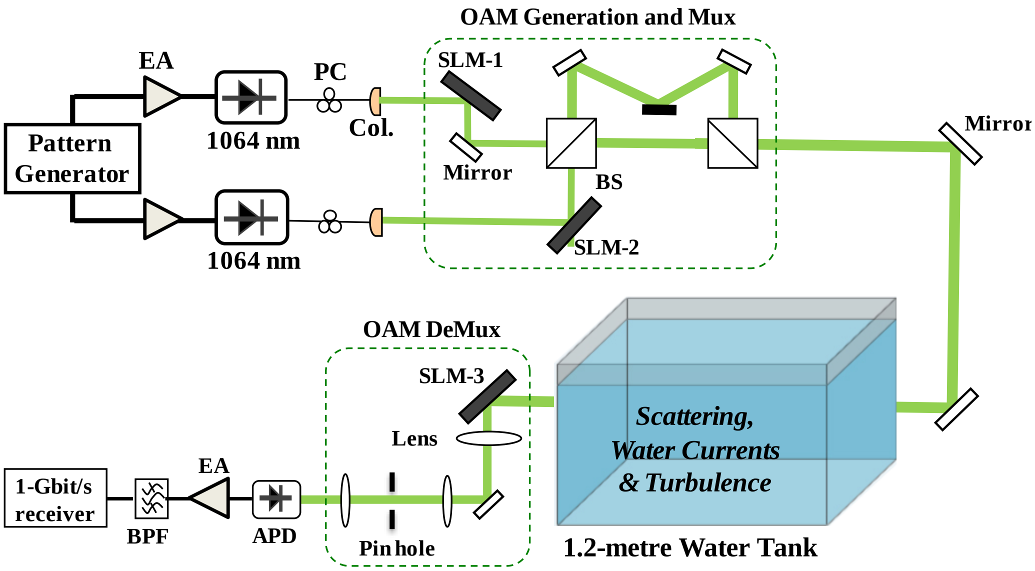

Supplementary Figure S2 | Experimental setup of a 4 Gbit/s OAM multiplexed underwater link using directly modulated 520-nm laser diodes. APD: avalanche photodiode. BS: beam splitter. Col.: collimator. DeMux: demultiplexing. EA: electric amplifier. Gen.: generation. LPF: low-pass filter. Mux: multiplexing. Mux: multiplexing. PC: polarization controller. SLM: spatial light modulator. TIA: transimpedance amplifier.

Supplementary Figure S3

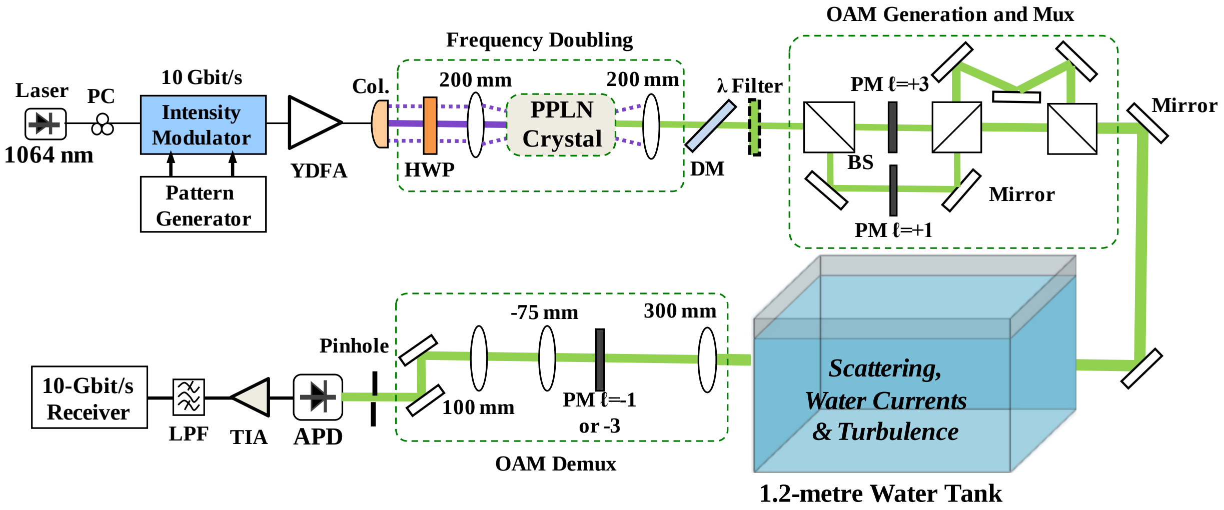

Supplementary Figure S3 | Experimental setup of a 40 Gbit/s OAM multiplexed underwater link using PPLN-based frequency doubling for signal generation. APD: avalanche photodetector. DM: dichroic mirror. HWP: half-wave plate. PM: metasurface phase mask. PPLN: periodically poled lithium niobate. YDFA: ytterbium-doped fibre amplifier.

Supplementary Figure S4

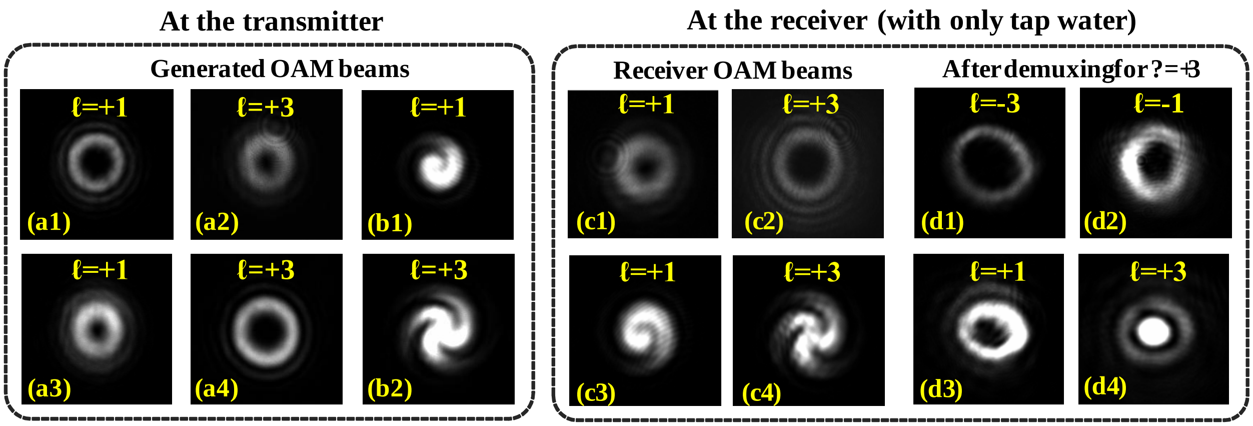

Supplementary Figure S4 | Captured intensity profiles at the transmitter and the receiver. (a) Intensity profiles of generated OAM beams ℓ = ±1 and ℓ = ±3. (b) Measured interferograms of generated OAM beams ℓ = +1 and +3. (c) Measured intensity profiles and interferograms of received OAM beams ℓ = +1, and ℓ = +3 after propagating through tap water. (d) Demultiplexed intensity profiles for transmitted OAM beam ℓ=+3 when a phase mask with an inverse spiral phase of either ℓ = -1, +1, -3 and +3 is used at the receiver.

Supplementary Figure S5

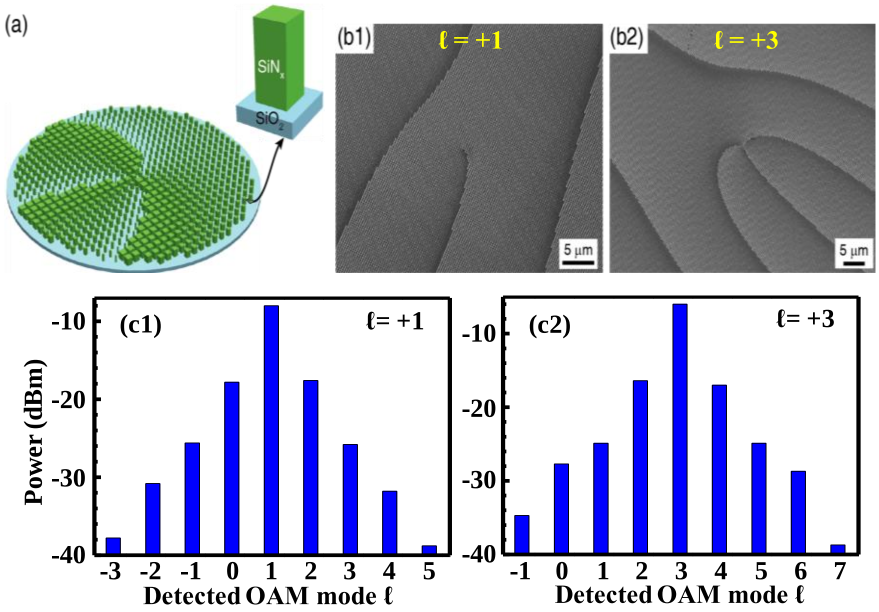

Supplementary Figure S5 | Dielectric metasurfaces phase mask for OAM generation and detection. (a) Schematic illustration of a blazed grating 'fork' phase mask for OAM ℓ=+3. (Inset) The building block of the metasurface structure: Square cross-section SiN_x nano-posts resting on a fused silica substrate. (b) Scanning electron micrographs of the fabricated phase masks of OAM ℓ=+1 and ℓ=+3. (c) Measured OAM power spectrum of the generated OAM beams with ℓ=+1 and +3.
